# Cdc42 activity in the trailing edge is required for persistent directional migration of keratinocytes

**DOI:** 10.1091/mbc.E23-08-0318

**Published:** 2023-11-01

**Authors:** Rutuja Patwardhan, Suchet Nanda, Jessica Wagner, Tom Stockter, Leif Dehmelt, Perihan Nalbant

**Affiliations:** a Department of Molecular Cell Biology, Center of Medical Biotechnology, University of Duisburg-Essen, 45141 Essen, Germany; b TU Dortmund University, Fakultät für Chemie und Chemische Biologie, 44227 Dortmund, Germany; Johns Hopkins University and National University of Singapore

## Abstract

Fibroblasts migrate discontinuously by generating transient leading-edge protrusions and irregular, abrupt retractions of a narrow trailing edge. In contrast, keratinocytes migrate persistently and directionally via a single, stable, broad protrusion paired with a stable trailing-edge. The Rho GTPases Rac1, Cdc42 and RhoA are key regulators of cell protrusions and retractions. However, how these molecules mediate cell-type specific migration modes is still poorly understood. In fibroblasts, all three Rho proteins are active at the leading edge, suggesting short-range coordination of protrusive Rac1 and Cdc42 signals with RhoA retraction signals. Here, we show that Cdc42 was surprisingly active in the trailing-edge of migrating keratinocytes. Elevated Cdc42 activity colocalized with the effectors MRCK and N-WASP suggesting that Cdc42 controls both myosin activation and actin polymerization in the back. Indeed, Cdc42 was required to maintain the highly dynamic contractile acto-myosin retrograde flow at the trailing edge of keratinocytes, and its depletion induced ectopic protrusions in the back, leading to decreased migration directionality. These findings suggest that Cdc42 is required to stabilize the dynamic cytoskeletal polarization in keratinocytes, to enable persistent, directional migration.

## Abstract (executive summary)

Cells migrate by coordinating dynamic cell shape changes in space and time. How this coordination enables cell-type specific migration dynamics is unclear. Here, the canonical cell protrusion regulator Cdc42 was investigated during cell migration.

In cell types that migrate less directionally, Cdc42 activity was localized at the leading-edge. In contrast, Cdc42 activity accumulated in the trailing edge of more directionally migrating keratinocytes.

Cdc42 was found to stabilize migration directionality by driving actin flow and myosin contraction. This reveals a keratinocyte-specific Cdc42 function that acts in the trailing edge, contrary to Cdc42’s; canonical function in cell protrusions.

## INTRODUCTION

Cell migration is a fundamental process that is essential for embryonic development, wound healing and the immune cell response. For efficient translocation and directional movement, most cell types form a polarized shape with actin-based protrusions in the front and a retracting tail (Ridley *et al.*, 2003). Depending on their specific requirements, distinct cell types migrate via variants of this general theme (Abercrombie *et al.*, 1970, Huttenlocher and Horwitz, 2011, Te Boekhorst *et al.*, 2016, Barros-Becker *et al.*, 2017, Nalbant and Dehmelt, 2018). Larger mesenchymal cells, such as fibroblasts, migrate much slower during wound healing, and generate transient protrusions in the front and myosin-based contractile stress fibers in their narrow back region (Friedl and Wolf, 2010, Huttenlocher and Horwitz, 2011). Basal keratinocytes migrate towards newly formed wounds with a high level of directionality (Bornes *et al.*, 2021). In vitro, this directional migration can be recapitulated via growth factor stimulation. In this assay, motile keratinocytes establish a distinct fan-shaped polarity with a large lamellipodium that covers around half of the cell periphery and a markedly broadened rear-end which contracts via fast acto-myosin based retrograde flow (Frank and Carter, 2004, Mohl *et al.*, 2012). Retaining this fan-shaped polarity is intimately linked to the persistent directional migration that is observed in this cell type (Frank and Carter, 2004).

Regulatory mechanisms that control the underlying cellular processes during migration, including protrusion, retraction, and adhesion, were studied extensively (Small and Resch, 2005, Zegers and Friedl, 2014). However, the fundamental principles how these processes are coordinated with each other in space and time are still poorly understood. The regulatory proteins of the Rho GTPase family, Rac1, Cdc42 and RhoA and their downstream effectors, are major regulators of the cytoskeleton and associated dynamic cell shape changes (Nobes and Hall, 1999, Zegers and Friedl, 2014, Ridley, 2015). Rac1 and Cdc42 induce actin protrusions called lamellipodia and filopodia, respectively, and RhoA controls Myosin-II motor mediated contractility in stress fibers and within the cell cortex. In addition, Cdc42 was linked to cell polarity and directional cell movement via multiple mechanisms (Etienne-Manneville, 2004, Macara, 2004, Farhan and Hsu, 2016). Due to their proposed antagonistic functions, classical front-back polarization models of migrating cells suggested mutually exclusive spatio-temporal activity dynamics of Rac1/Cdc42 in the front and RhoA in the back of individual cells. Studies with fluorescence based live-cell sensors suggest that such an antagonistic model might underly front-back segregation of Rho GTPase activities in small migratory cells, such as neutrophils (Wong *et al.*, 2006, Wong *et al.*, 2007, Tsai *et al.*, 2019). However, in larger fibroblasts and osteosarcoma cells, Rho activity dynamics were segregated within smaller cell regions, including the leading edge, and subcellular contractile nodes in central adhesive areas (Kraynov *et al.*, 2000, Nalbant *et al.*, 2004, Pertz *et al.*, 2006, Nalbant *et al.*, 2009, Graessl *et al.*, 2017). These observations suggest that multiple distinct regulatory mechanisms exist to control distinct Rho activity patterns.

Previous studies suggest that Rho GTPases also contribute to tissue homeostasis and regulate keratinocyte migration during wound healing in vivo and in vitro (Tscharntke *et al.*, 2007, Jackson *et al.*, 2011, Kiwanuka *et al.*, 2016), however, the underlying molecular mechanisms are still poorly understood. Here, we identified Cdc42 as a major regulator of persistent cell polarity and directional motility in keratinocytes. Our studies show that Cdc42 is required to increase and stabilize retrograde flow of actin and myosin at the trailing edge of these cells, and thereby controls their persistent front-back polarization. Thus, in contrast to its role at the leading edge of mesenchymal cells, Cdc42 stabilizes the persistent, directional cell migration of keratinocytes by controlling acto-myosin dynamics in their rear-end.

## RESULTS

**FIGURE 1:**
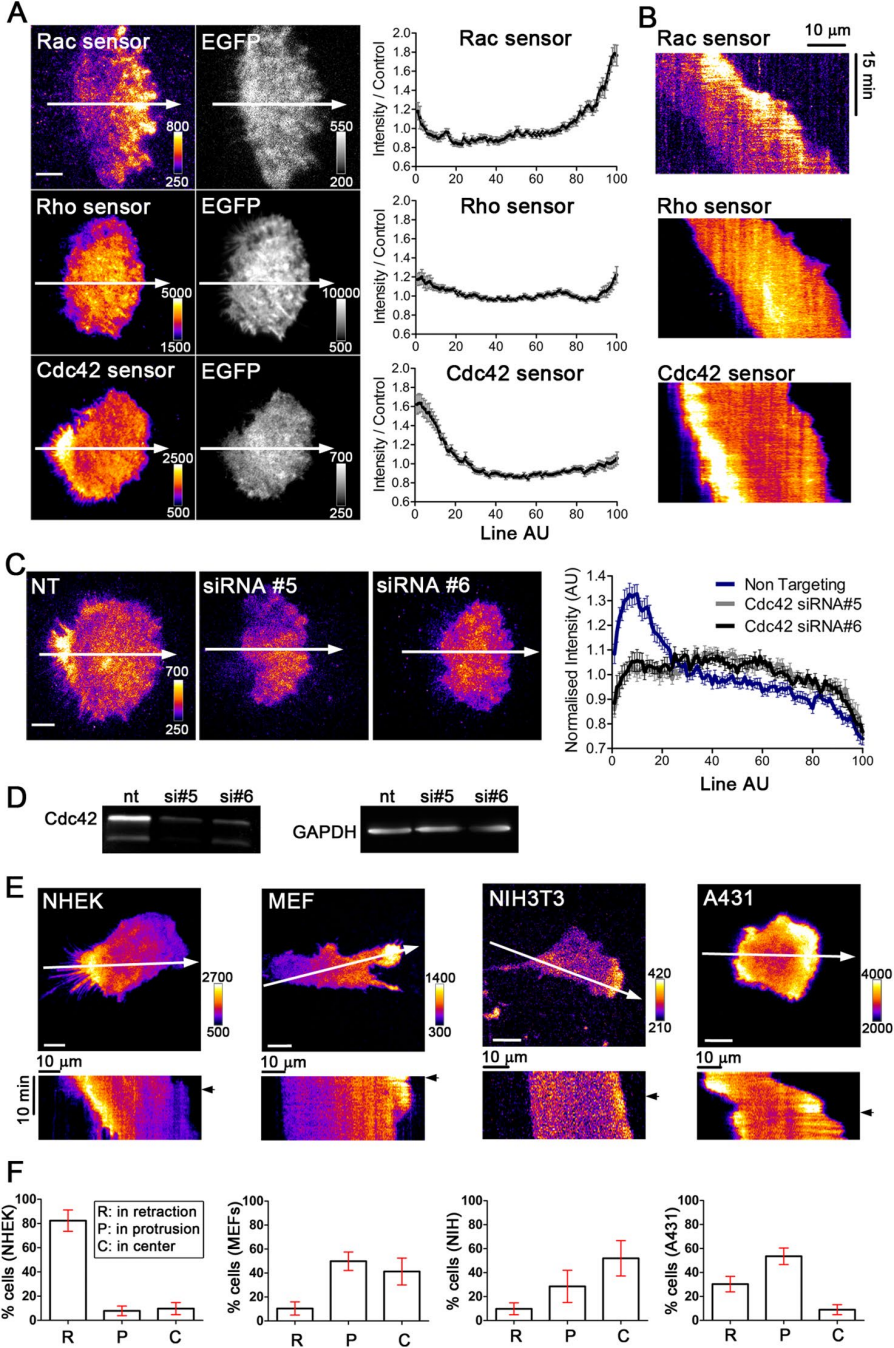
Distinct spatio-temporal dynamics of Rho GTPase activity in motile keratinocytes. (A) Left: Representative TIRF images of normal human epidermal keratinocytes (NHEK) coexpressing EGFP control plasmid with activity sensors for Rac (mCherry-3X-p67^phox^-GBD), Rho (mCherry-Rhotekin-GBD) or Cdc42 (mCherry-WASP-GBD), respectively. White arrows correspond to the cell migration direction. Right: Average sensor intensity profiles along the arrows shown in corresponding left panels. *n* > 25 cells from 3 experiments. (B) Kymographs corresponding to arrows in A. Scale bar: 10 µm. (C) Cdc42 depletion leads to loss of trailing edge sensor signal. Left: TIRF images of the Cdc42 activity sensor in NHEK cells treated with non-targeting siRNA or two distinct siRNAs that target Cdc42. Right: Average sensor intensity profiles along the arrows that point in the cell migration direction (left). *n* > 29 cells from 3 experiments. (D) Representative Western blot to quantify siRNA-mediated Cdc42 knockdown. (E) Distinct cell type–specific Cdc42 activity patterns. Upper panels: Representative images of the Cdc42 activity sensor in different cell types. White arrows point in the cell migration and/or cell protrusion direction. Lower panels: Kymographs that correspond to white arrows drawn in the upper images. Black arrows correspond to the time point of the image shown above. (F) Classification of the predominant Cdc42 activity patterns in individual cells. Three mutually exclusive categories were considered, in which the majority of Cdc42 signals was enriched: protrusions (P), retractions (R) or the cell center (C). *n* > 29 cells from 3–4 experiments. Scale bars: 10 µm. Error bars represent S.E.M.

To gain insight into the underlying mechanisms that control the distinct persistent polarized morphology and directional migration of normal human epidermal keratinocytes (NHEKs), we investigated the spatio-temporal activity dynamics of the best-studied Rho GTPases Rac1, Cdc42 and Rho. To measure the activity state of the endogenous GTPases, we used TIRF microscopy and translocation sensors that are based on fluorescent protein coupled effector domains. To measure Rac1 activity, we recently generated an improved Rac1 sensor (Nanda *et al.*, 2023), which was based on a previously published p67^phox^ GTPase binding domain (GBD) construct (Graessl *et al.*, 2017). The improved sensor contains three tandem repeats of the p67^phox^ GBD attached to the red fluorescent protein mCherry. To measure Rho and Cdc42 activity, we employed established Rhotekin-GBD and WASP-GBD-based sensors (delCMV-mCherry-Rhotekin-GBD and delCMV-mCherry-WASP-GBD) respectively (Graessl *et al.*, 2017). Similar to prior studies in other migratory cell types such as fibroblasts, Rac1 activity was localized to protrusions at the leading edge (Figure 1A,B and Supplemental Movie S1) (Kraynov *et al.*, 2000, Pratt *et al.*, 2005, Machacek *et al.*, 2009). Interestingly, we did not detect elevated Rho activity signals in the retracting trailing edge (Figure 1A and Supplemental Movie S1). This finding was unexpected as the local retrograde flow of actin and Myosin-II drives contraction in this region, and active Rho is best known for its role in stimulating Myosin-II activity (Riento and Ridley, 2003).

**Figure:** Movie S1 **Spatio-temporal Rho GTPase activity dynamics in normal human epidermal keratinocytes (NHEKs)**. Time-lapse TIRF videos of the activity sensors for Rac (mCherry-3X-p67^phox^-GBD), Rho (mCherry-Rhotekin-GBD) and Cdc42 (mCherry-WASP-GBD) in representative motile keratinocytes. Images were collected with a frame rate of 3/min. Scale bar, 10 μm.

Surprisingly, we found highly elevated Cdc42 activity in the keratinocyte trailing edge. As shown in Figure 1A,B, the local trailing edge enrichment of Cdc42 activity in keratinocytes was precisely aligned with the direction of migration (see also Supplemental Movie S1). Cdc42 depletion via two distinct siRNAs abolished the elevated Cdc42 activity sensor signal, confirming the specificity of the sensor measurements (Figure 1C,D and Supplemental Movie S2).

**Figure:** Movie S2 **Cdc42 depletion leads to reduced trailing edge activity signal**. TIRF videos of the activity sensor for Cdc42 (mCherry-WASP-GBD) in representative motile keratinocytes treated with nontargeting siRNA or two distinct siRNAs that target Cdc42 (siRNA5/6). Images were collected with a frame rate of 3/min. Scale bar, 10 μm.

Motile keratinocytes retract their back via a strong acto-myosin retrograde flow, and this persistent contraction is thought to be important for maintaining persistent directional migration. In contrast, other migratory cell types such as fibroblasts use a lamellipodia-driven migration mode with strong protrusion dynamics in the front and relatively passive bundles of stress fibers in the back (Ridley *et al.*, 2003, Huttenlocher and Horwitz, 2011). In previous studies of fibroblasts that employed a sensor based on ratiometric measurements of dyes with distinct environmental sensitivity, Cdc42 activity was found to be localized in dynamic, nascent protrusions at the leading edge, similar to active Rac1 (Nalbant *et al.*, 2004, Hanna *et al.*, 2014). Using the translocation sensor approach, we confirmed these previous findings in two fibroblast cell types (MEFs and NIH3T3) (Figure 1E,F and Supplemental Movie S3). Here, Cdc42 activity was absent from the retracting tail regions, suggesting that the spatio-temporal activity dynamics of Cdc42 in motile keratinocytes is distinct from activity dynamics in fibroblasts (Figure 1E). In the A431 epidermoid carcinoma cell line, which is derived from human keratinocytes, Cdc42 activity partly corresponded to keratinocytes and fibroblasts, and was elevated both during cell protrusion and retraction phases (Figure 1E,F and Supplemental Movie S3).

**Figure:** Movie S3 **Distinct cell type specific Cdc42 activity patterns**. Time-lapse TIRF videos of the Cdc42 activity sensor (mCherry-WASP-GBD) in different cell types. Images were collected with a frame rate of 3/min. Scale bars, 10 μm.

**FIGURE 2:**
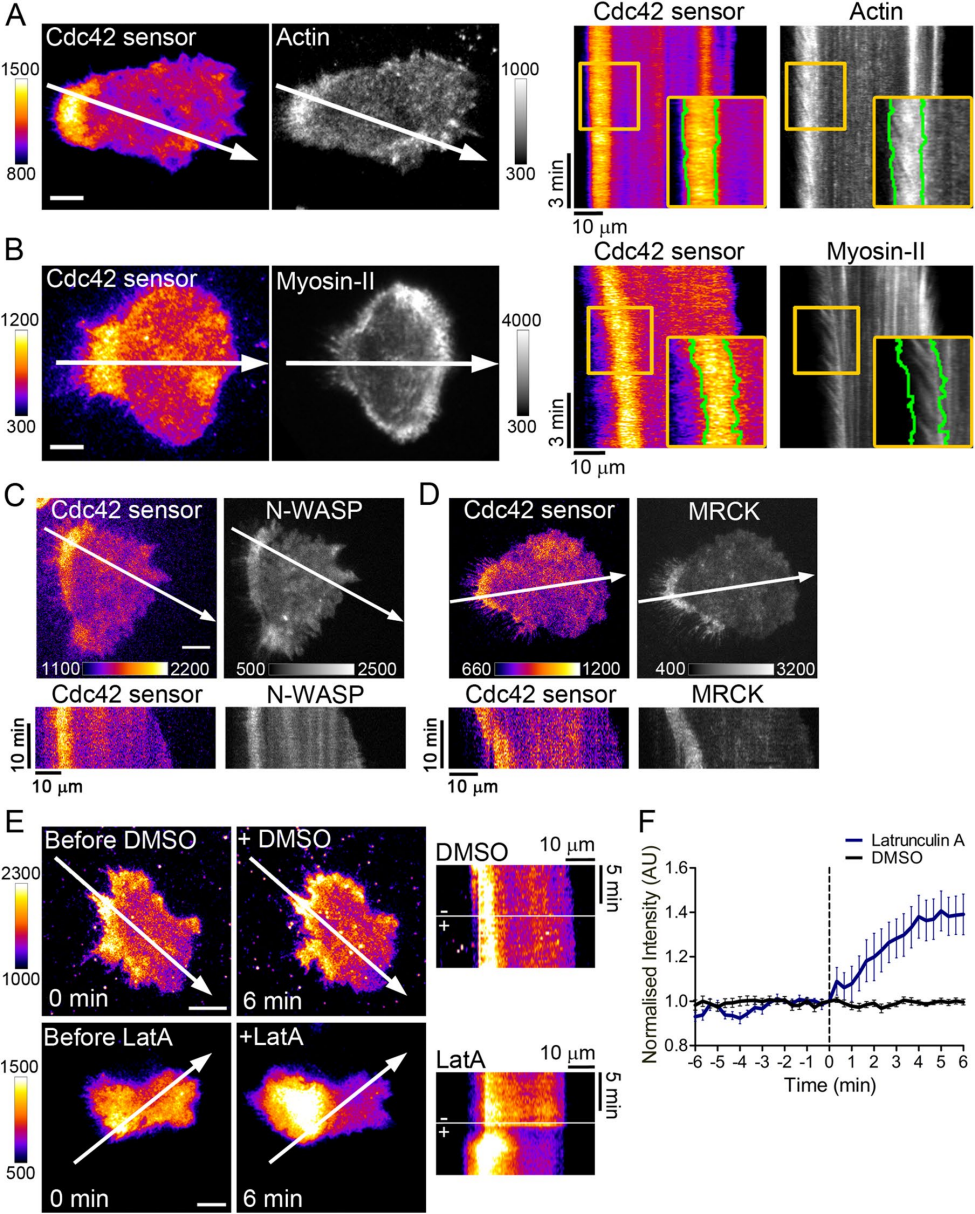
(A and B) Cdc42 trailing edge activity colocalizes with acto-myosin retrograde flow. Left: First time-points of representative TIRF videos of NHEK cells coexpressing the Cdc42 activity sensor and (A) Actin (EGFP-Actin) or (B) Myosin-II (GFP-NMHCIIa). White arrows point in the direction of cell migration. Right: Kymographs along the white lines in left panels, showing local Cdc42 activity patterns and the flow of (A) actin and (B) Myosin-II, respectively. (C, D) Cdc42 activity colocalizes with its effectors N-WASP and MRCKbeta at the trailing edge of keratinocytes. Top panels: First time-points of representative TIRF videos of NHEK cells coexpressing the Cdc42 activity sensor and (C) full-length N-WASP (GFP-N-WASP) or (D) MRCKbeta (pEGFP-N1-Cdc42BPB). Bottom panels: Kymographs along the white lines in corresponding top panels (see also Supplemental Movies S5 and S6). Scale bars: 10 μm. *n* > 50 cells (3–5 experiments). (D and F) Inhibition of actin dynamics leads to increase and delocalization of Cdc42 activity at the trailing edge. (E) Left panels: Representative images of cells expressing the Cdc42 activity sensor, before (t = 0 min) and after (t = 6 min) treatment with vehicle control (DMSO) or Latrunculin A (10 µM), respectively. Scale bars: 10 μm. Middle panels: Kymographs correspond to white lines in the images in left panels. (F) Signal intensity dynamics of the Cdc42 activity sensor at the trailing edge during DMSO or Latrunculin A treatment, quantified via kymographs shown in e (see Methods for details). Mean signal intensities were background corrected and normalized to the time point right before drug or vehicle addition (*n* > 27 cells from 3 independent experiments). Error bars represent SD.

Elevated activity of Cdc42 coincided with two distinct subregions in keratinocytes: the trailing edge of the cell, at which new actin filaments are polymerized (Theriot and Mitchison, 1991; Figure 2A and Supplemental Movie S4) and more proximal regions, in which Myosin-II incorporates into these filaments (Lin *et al.*, 1996, Svitkina *et al.*, 1997; Figure 2B and Supplemental Movie S4). It is well established that Cdc42 stimulates actin polymerization (Rohatgi *et al.*, 1999, Lammers *et al.*, 2008, Kage *et al.*, 2017, Mosaddeghzadeh and Ahmadian, 2021). However, some studies have shown that Cdc42 can also stimulate Myosin-II activity (Leung *et al.*, 1998, Wilkinson *et al.*, 2005). We therefore investigated potential roles of specific Cdc42 effectors that might stimulate either actin polymerization or Myosin-II activation. In these studies, we were able to detect increased levels of Cdc42 activity at the trailing edge in about 80–90% of all cells. In 65 ± 4% of those cells, we also detected increased levels of the actin nucleation promoting factor Neural Wiskott-Aldrich syndrome protein (N-WASP; Gene name: WASL; Rohatgi *et al.*, 1999) co–localized with active Cdc42 at the trailing edge. This co–localization was even more pronounced for the myosin activating myotonic dystrophy-related Cdc42-binding kinase beta (MRCKbeta; Gene name: CDC42BPB; Leung *et al*.,1998, Wilkinson *et al*., 2005) (98 ± 3%) (Figure 2C,D and Supplemental Videos S5 and S6). By activating these two distinct effector-mediated functions, Cdc42 might be able to maximally stimulate actin retrograde flow, which is known to be dependent on a combination of pushing forces that are generated by actin polymerization at the leading edge and pulling forces that are generated by myosin motor driven contractility (Medeiros *et al.*, 2006).

**Figure:** Movie S4 **Cdc42 trailing edge activity colocalizes with acto-myosin retrograde flow**. TIRF videos of cells co-expressing the Cdc42 activity sensor (mCherry-WASP-GBD) and Actin (EGFP-Actin) or Myosin-II (GFP-NMHCIIa), respectively. Images were collected with a frame rate of 20/min. Scale bars, 10 μm.

**Figure:** Movie S5 **Cdc42 trailing edge activity colocalizes with N-WASP**. TIRF videos of cells co-expressing the Cdc42 activity sensor (mCherry-WASP-GBD) and GFP-N-WASP. Images were collected with a frame rate of 3/min. Scale bars, 10 μm.

**Figure:** Movie S6 Cdc42 trailing edge activity colocalizes with MRCKbeta. TIRF videos of cells coexpressing the Cdc42 activity sensor (mCherry-WASP-GBD) and EGFP-MRCKbeta. Images were collected with a frame rate of 3/min. Scale bars, 10 μm.

Previous studies suggested that spatio-temporal activity dynamics of Rho GTPases are dependent on positive feedback mechanisms, which involve effectors that can also directly or indirectly activate the corresponding GTPase, for example via recruiting a GEF (Wang *et al.*, 2002, Srinivasan *et al.*, 2003, Bement *et al*., 2015; Graessl *et al.*, 2017, Nalbant and Dehmelt, 2018). As many Cdc42 effectors are actin regulatory proteins, we investigated if actin dynamics might play a role in such a potential feedback mechanism in keratinocytes. Surprisingly, inhibition of actin polymerization by latrunculin A treatment significantly broadened the area of Cdc42 activity in the trailing edge (Figure 2E, right panel and Supplemental Movie S7). Furthermore, the Cdc42 activity signal in this region was significantly increased upon latrunculin A treatment. This suggests that actin dynamics rather play an inhibitory role in controlling Cdc42 activity patterns (Figure 2F).

**Figure:** Movie S7 **Inhibition of actin dynamics leads to increase and delocalization of Cdc42 activity in the trailing edge**. Cells expressing the Cdc42 activity sensor were treated with vehicle control (DMSO) or Latrunculin A (10 μM), respectively, at t = 0 min. Frame rate: 3/min. Scale bar, 10 μm.

**FIGURE 3:**
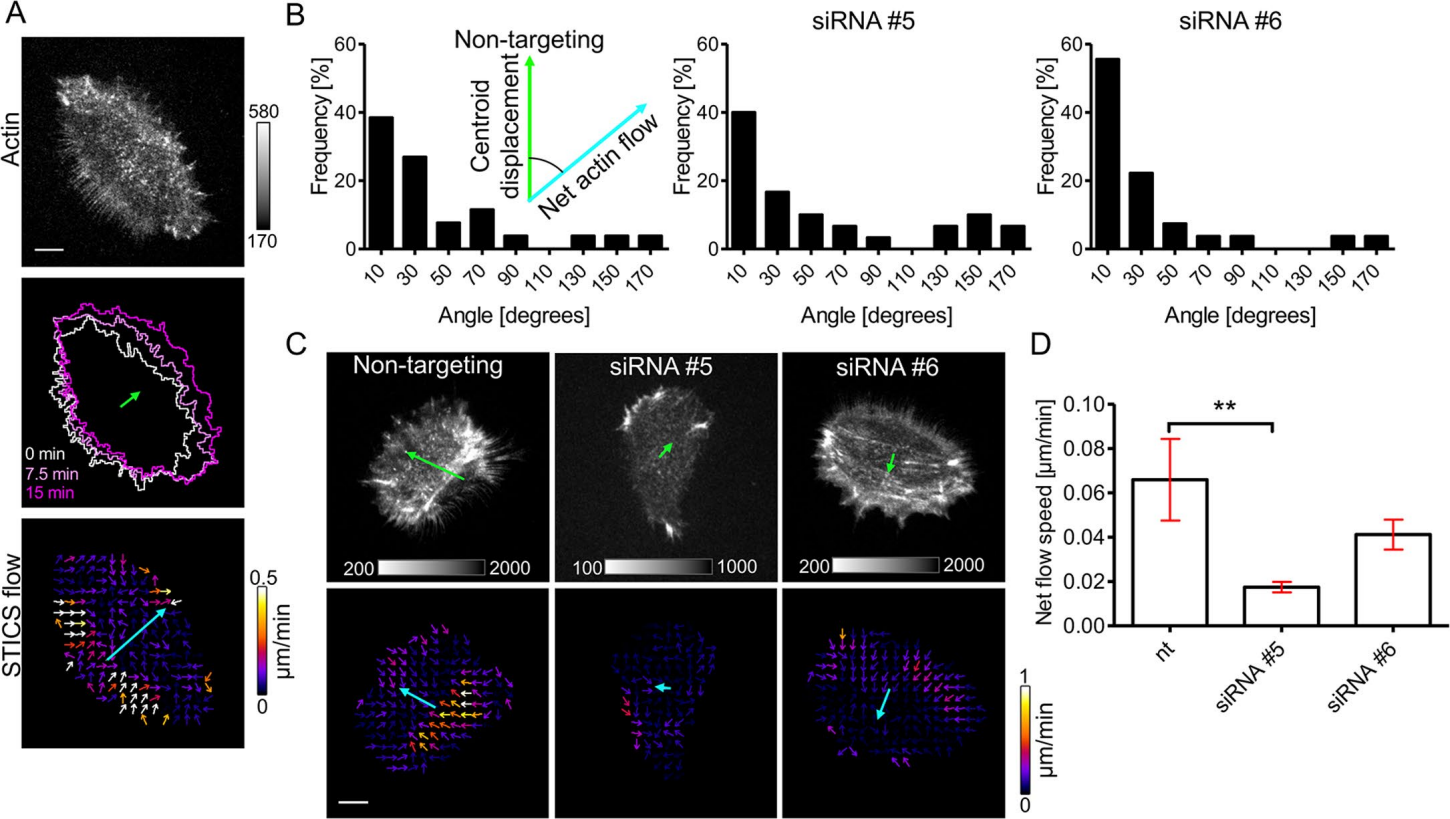
Depletion of Cdc42 in keratinocytes decreases polarized acto-myosin flow. (A and C) Upper panels: Representative TIRF images of control (A) or siRNA-treated cells (C) that express EGFP-Actin. Lower panels: Color-coded STICS flow vector fields depicting actin movement velocities corresponding to upper panels. The green arrows represent the displacement of the cell centroid over 15 min, the cyan arrows represent the net flow direction and speed. (A) Middle Panel: cell outlines at the specified time points. (B) Histograms of the distributions of the angle between the displacement and net flow vectors of control (non-targeting) and Cdc42 depleted cells (siRNA 5/6) (*n* > 26 cells from 3 independent experiments). (D) Column plot of the magnitude of the net flow velocity vectors, corresponding to the length of cyan arrows shown in (C). Scale bars: 10 μm. Error bars represent S.E.M; Analyzed with One Way ANOVA and Dunnett’s Multiple comparison Test.

Furthermore, we investigated, how Cdc42 activity at the trailing edge affects cytoskeletal dynamics in migrating keratinocytes. We used spatiotemporal image correlation spectroscopy (STICS) to measure the flow velocity of acto-myosin structures relative to the cell substrate. This analysis showed that the majority of flow occurred near the trailing edge of cells (Figure 3A). We were also able to extract a net flow direction, which pointed to a similar direction as the centroid of the migrating cell body (Figure 3A). This suggests that the polarized asymmetry of the acto-myosin flow is coupled to the direction of keratinocyte migration. In the majority of cells, Cdc42 knockdown did not affect this coupling (Figure 3B), however, the net acto-myosin flow magnitude was substantially reduced (Figure 3C,D and Supplemental Movie S8). This shows that Cdc42 activity is required to maintain the robust, front-back polarized asymmetry of acto-myosin retrograde flow in migrating keratinocytes.

**Figure:** Movie S8 **Depletion of Cdc42 in keratinocytes decreases polarized acto-myosin flow**. Representative TIRF movies of control (non-targeting) or Cdc42 depleted cells (Cdc42 siRNA #5/6) that express EGFP-Actin (upper panels). Lower panels: Color-coded STICS flow vector fields depict actin movement velocities corresponding to upper panels. Frame rate: 12/min. Scale bar, 10 μm.

**FIGURE 4:**
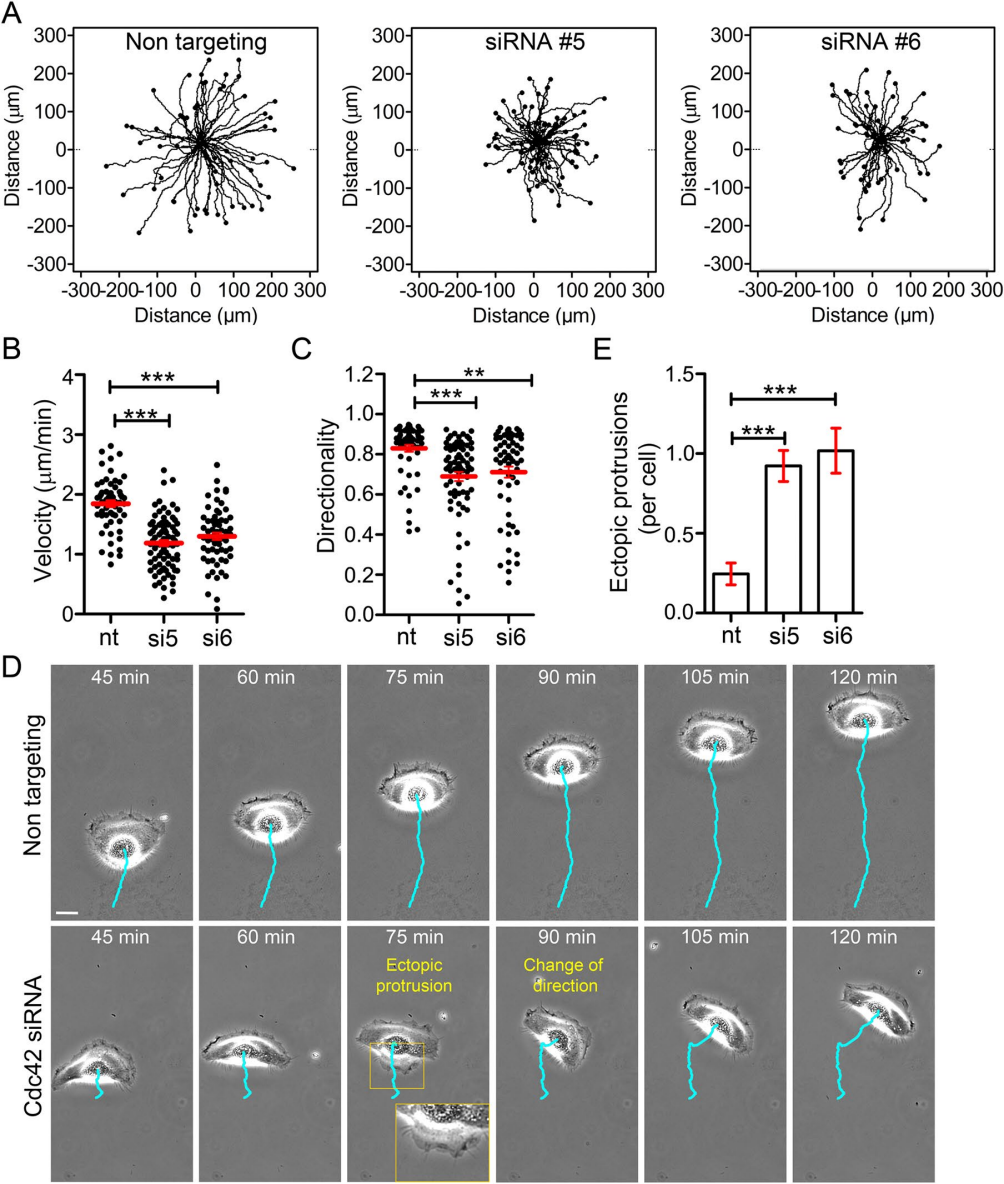
Cdc42 suppresses ectopic protrusions at the trailing edge of keratinocytes and is essential for directional cell migration. (A) Migration tracks of NHEK cells treated with nontargeting siRNA (nt) or siRNAs that target Cdc42 (siRNA #5/#6). Cell tracking was performed via phase contrast video microscopy. Frame-rate: 1/min, *n* > 61 cells from 3 experiments each. (B and C) Quantification of migration velocity and directionality derived from data shown in A. (D) Morphology of a representative siRNA treated keratinocyte. The cell trajectory is represented by a cyan line. Scale bar: 20 μm. (E) Average number of ectopic protrusions generated at the trailing edge of migrating keratinocytes. Error bars represent S.E.M; Analyzed with One-Way ANOVA and Dunnett’s Multiple comparison Test.

Next, single cell tracking experiments revealed that both migration velocity and directionality were significantly reduced in cells lacking Cdc42 (Figure 4A–C). Morphodynamic analyses of individual cells also revealed a significant change in cell protrusion dynamics upon Cdc42 depletion. Control keratinocytes persistently maintained a front-back polarized morphology that was aligned to the direction of cell migration. This morphology was characterized both by a persistent, protruding front and a persistent, retracting back (Figure 4D and Supplemental Movie S9). In contrast, cells depleted of Cdc42 were not able to maintain this persistent back retraction and instead frequently generated cell protrusions at the trailing edge which often led to a change of migration direction (Figure 4D,E and Supplemental Movie S9).

**Figure:** Movie S9 **Cdc42 depletion decreases directional migration of keratinocytes**. Phase contrast videos of NHEK control (non-targeting) or Cdc42 depleted cells (Cdc42 siRNA #6). Coloured lines represent migration tracks starting at t = 0 min. Frame rate: 1/min.

The dynamic control of trailing edge retraction in keratinocytes is a complex process that involves the robust actin retrograde flow and the spatio-temporal coordination of this process with dynamic turn-over of flanking adhesion sides (Mohl *et al.*, 2012). Here, we reveal that Cdc42 is a key regulator of this important function of keratinocytes. We show that localized activity of Cdc42 in keratinocytes surprisingly co–localized with the retracting, trailing back region of cells, which is in contrast to previous studies that reported active Cdc42 in the protrusive front. In keratinocytes, the retracting back region is characterized by a robust acto-myosin flow that is aligned with the direction of the migration axis, and we found that Cdc42 is critical for generating and maintaining this structure. Cdc42 could perform this function by stimulating both an actin polymerization based “push” force via activating the nucleation promoting factor N-WASP and formins (Rohatgi *et al.*, 1999, Lammers *et al.*, 2008, Kage *et al.*, 2017) and a contractile “pull” force by activating the myotonic dystrophy kinase-related CDC42-binding kinase MRCK (Leung *et al.*, 1998, Wilkinson *et al.*, 2005) that stimulates Myosin-II activity. Interestingly, Cdc42 does not only act upstream of actin, but instead is also inhibited by actin (Figure 2B–C). This closes a negative feedback loop, which might play a role in restricting Cdc42 activity at the trailing edge.

A key insight from our studies is that distinct spatio-temporal Rho GTPase activity dynamics underlie cell-type specific migration behavior. Typical mesenchymal cells, which frequently change their movement direction by generating highly dynamic cycles of cell protrusion and retraction show elevated activities of all three major Rho proteins at the leading-edge (Machacek *et al.*, 2009). In contrast, keratinocytes generate a stable polarized morphology and persistent directionality, which is dependent on the stable presence of active Cdc42 at the trailing edge. Such fundamental differences in spatio-temporal organization and coordination of Rho GTPase activities might require distinct sets of regulators, that are differentially expressed in a cell-type specific manner.

In conclusion, our findings show that Cdc42 controls the spatio-temporal dynamics of the acto-myosin cytoskeleton at the trailing edge of motile keratinocytes, and that this role is intimately linked to the distinct migratory mode used by this cell system. In contrast to the lamellipodia-driven migration of mesenchymal cells, in which the tail retraction appears rather abrupt and often appears to happen rather passively due to the release of adhesions, the persistent, polarized acto-myosin flow that is aligned with the migration axis facilitates the typical persistent, directional gliding migration mode of keratinocytes (Figure 4D). Previous in vivo studies showed that during wound healing and re-epithelialization, individual epidermal keratinocytes migrate with prominent directionality towards the wound bed (Bornes *et al.*, 2021). Via controlling the robust polarized cell shape and steady directionality, Cdc42 might thereby promote efficient migration of keratinocytes to facilitate effective wound healing in skin tissues.

## MATERIALS AND METHODS

Request a protocol through *Bio-protocol*.

### Constructs and siRNAs

Sensors to measure Rho (delCMV-mCherry-Rhotekin-GBD) and Cd4c2 activity (delCMV-mCherry-WASP-GBD) (Graessl *et al.*, 2017) and the delCMV-mCherry-actin construct (Watanabe and Mitchison, 2002, Schulze *et al.*, 2014, Graessl *et al.*, 2017) as well as the generation and characterization of the Rac1 activity sensor (delCMV-mCherry-3X-p67^phox^-GBD) (Nanda *et al.*, 2023) containing three tandem repeats of the p67^phox^-GBD were described previously. GFP-NMHCIIA ([Wei and Adelstein, 2000], plasmid 11347), EGFP-MRCKbeta (EGFP-Cdc42BPB) ([Ando *et al.*, 2013], plasmid 50759) and GFP-N-WASP ([Hussain *et al.*, 2001], plasmid 47406) were obtained from Addgene. For Cdc42 depletion, RNAi Max together with either ON-TARGET plus nontargeting siRNA#2 (UGGUUUAC­AUGUUGUGUGA) or ON-TARGET Plus Cdc42 siRNA#5 (CGGAAUAUGUACCGACUGU) and #6 (GCAGUCACAGUUAUGAUUG) (all Dharmacon) was used.

### Cell culture and reagents

Normal human epidermal keratinocytes neonatal (NHEK; FC-0007, Lifeline Cell Technology, MD USA, CellSystems, Troisdorf, Germany) were cultured in DermaLife basal medium supplemented with DermaLife K LifeFactors kit (Lifeline Cell Technology, Frederick, MD, USA). During cell splitting, the TrypKit (CellSystems, Troisdorf, Germany) was used for trypsinization. Only cells up to passage number 5 were used for experiments. Mouse embryonic fibroblasts (MEFs), NIH3T3 and A431 cells were maintained in DMEM (GlutaMAX Life technologies, Life Technologies or PAN-Biotech) supplemented with 10% FBS (Life technologies, Life Technologies). For A431 cells, the medium was supplemented with 1% Penicillin-Streptomycin (PAN-Biotech) and 1% L-Glutamine (PAN-Biotech). Cells were maintained at 37°C and in 5% CO_2_ humidified atmosphere. For live cell imaging, NHEK, MEFs and NIH3T3 were plated on fibronectin (10 µg/ml, 15 min, Corning) coated glass dishes (MatTek) and transfected after 24 h of seeding. Imaging was performed on the next day after transfection. For cell transfection, either TransIT (Mirus) (NHEK) or Lipofectamine 3000 (Invitrogen) (A431) or Lipofectamine 2000 (Invitrogen) (MEFs and NIH3T3) was used. A431 cells were replated on fibronectin (10 µg/ml, 4°C, overnight, Merck) coated glass dishes (MatTek) 1 day after transfection and allowed to adhere for 1 h before imaging. Imaging was performed in Hanks balanced Salt solution (HBSS, Life technologies, Life Technologies) with 1 mM of HEPES (Life technologies, Life Technologies) (MEFs and NIH3T3), Dermalife Basal media with supplements and 1–2 mM of HEPES (Life technologies, Life Technologies) (NHEK) or in DMEM (PAN-Biotech with 1 mM HEPES) (A431). For cell migration, NHEKs were stimulated with recombinant human EGF (100 ng/ml, R&D Systems) for 1 h before imaging. On stage addition of Latrunculin A (5–10 µM, Sigma) or corresponding vehicle control (DMSO; 1:1000 dilution) was used to study acute effects of actin disruption.

All ON-TARGET plus siRNAs (nontargeting control [UGGUUUACAUGUUGUGUGA], Cdc42 siRNA #5 [CGGAAUAUGUACCGA­CUGU], Cdc42 siRNA #6 [GCAGUCACAGUUAUGAUUG]) were purchased from GE Healthcare Dharmacon. Cells were transfected with 20 nM of the respective siRNAs using the transfection reagent Lipofectamine RNAiMax (Invitrogen) and incubated for 24 h followed by cell splitting and seeding for experiments as required. All measurements were performed 72 h post siRNA treatment of cells. Knockdown efficiency was determined by Western blot analysis.

### Western blot analysis

Cells were washed once with ice-cold PBS and lysed in ice-cold RIPA buffer (50 mM Tris, pH 7.5, 150 mM NaCl, 1% NP-40, 0.25% sodium deoxycholate, 1 mM EDTA, 1 × protease inhibitor cocktail, and 1 × phosphatase inhibitor cocktail). Cell debris were removed by centrifugation at 14,000 g for 15 min at 4°C. Protein concentration in the supernatants was determined via Bradford assay. Equal amounts of total protein were mixed with 5 × Laemmli sample buffer, boiled at 95°C for 10 min and separated by SDS–PAGE. After electrophoresis, proteins were transferred on a PVDF membrane using a semidry blotter. Blots were blocked for 60 min at RT with 5% BSA in TBS-T (20 mM Tris, pH 7.6, 137 mM NaCl, and 0.1% Tween-20) and incubated overnight at 4°C with a primary antibody that recognizes Cdc42 (1:100 dilution; sc-8401; SantaCruz) in blocking solution. Membranes were washed three times with TBS-T and incubated with the HRP-conjugated antimouse secondary antibody (1:20,000) for 1 h at RT. After additional washing steps with TBS-T and TBS (20 mM Tris, pH 7.6, and 137 mM NaCl) proteins were visualized with ECL Western blotting substrate (SuperSignal West Pico, Thermo Scientific).

### Microscopy

The majority of total internal reflection fluorescence microscopy (TIRF-M) experiments was performed using an Eclipse Ti-E (Nikon) inverted microscope equipped with an AOTF laser modulator and Combiner (excitation laser lines: 488 nm and 561 nm), a TIRF illuminator Unit, a perfect focus system (PFS), a laser dual spinning disk scan Head (CSU-X1: Yokogawa), and an iXon3 897 single-photon-detection EMCCD camera (16*16 µm Pixels, EM gain 50–100, 2*2 or 1*1 Binning). Images were acquired using an Apo TIRF 100X/1.49 NA or Apo TIRF 60X/1.49 NA oil immersion objective, CSU Quad Dichoric mirror set (Andor technologies, Yokogawa CSU-X1) and a TIRF Dual line Beamsplitter zt 488/561 rpc (ANDOR iXON DU-897). Acquisition was controlled with the Andor IQ Software. Additional TIRF experiments were performed using a Ti2-E (Nikon) inverted microscope equipped with 4 color TIRF, H-TIRF for fully automated TIRF adjustments, 2X iXon Life DU-888 back illuminated EMCCD cameras (13*13 µm pixels; Andor Technology iXon Life DU-888), omicron Light Hub-4 compact laser beam contributor with AOTF laser modulator, diode laser cw LuXX 488 nm (250mW) and DPSS laser cw Jive 561 nm (300mW). The specifications for the quadband dichroic and the emission filters for TIRF were as follows: TIRF Emission Filter#1, EGFP, 525/50 nm; TIRF Emission Filter #2, RFP, 600/50 nm; NSTORM QUAD (Excitation dichroic 497–553 nm, Emission 523.5/42 nm; Excitation dichroic 575–628 nm, Emission 603/44 nm). Images were acquired with a CFI Apochromat TIRF 60x/1.49 oil immersion objective using the Nikon Imaging Software NIS elements Advanced Research. Imaging of A431 cells was performed on an Olympus IX-81 microscope, equipped with a TIRF-MITICO motorized TIRF illumination combiner, an Apo TIRF 60 × 1.45 NA oil immersion objective and a ZDC autofocus device. Imaging was performed using a quadruple bandpass dichroic mirror (U-M3TIR405/445/514/561, Olympus, Hamburg) in combination with a Semrock Brightline emission filter (HC 629/53, AHF Analysentechnik, Tübingen) and a 561 nm CellR diode laser (100 mW). For detection, an EMCCD camera was used at medium gain without binning and images were acquired using the Cell^R Software (Olympus). Phase contrast video microscopy images were acquired on a Nikon-Ti-E inverted microscope with a CoolSNAP Hq2 interline-transfer CCD camera (Roper Scientific) and a CFI Plan Fluor ELWD 20x/0.45 DIC objective. Images were acquired with the Nikon NIS Elements AR software.*

### Image processing and data analysis

All images were processed using ImageJ/Fiji Software (Schindelin *et al.*, 2012). To correct for lateral drift, the image stabilizer plugin was used (K. Li, “The image stabilizer plugin for ImageJ,” http://www.cs.cmu.edu/∼kangli/code/Image_Stabilizer.html, February, 2008). Unless noted otherwise, further image manipulations were restricted to adjustment of brightness levels, cropping, scaling and false color-coding via look-up tables.

To quantify the distribution of Rac, Rho and Cdc42 activity intensity in front-back polarized cells (Figure 1A), time lapse sequences were first obtained to determine the direction of migration. A representative image was then chosen for each time lapse sequence, and the signal intensity was measured along a line that corresponded to the migration direction, and that started at the retracting and ended at the protruding cell edge. The signal intensity was normalised to the average intensity along the entire line, and the position along the cell axis was normalized to the total length of the line. Finally, the GTPase sensor signal intensities were normalised to control signal intensities (cytoplasmic EGFP) for each cell. For Cdc42 depletion experiments (Figure 1C) this last normalization step was not necessary as relative measures of Cdc42 versus control siRNA treatments were sufficient. Therefore, this last normalization was omitted for these experiments.

Classification of Cdc42 activity maxima in migrating cells (Figure 1E) was based on time lapse image sequences. Signal intensities were measured in protrusions, retractions and central cell attachment areas via ImageJ measure plugin to determine, in which of these cell structures the Cdc42 activity sensor signal was maximal.

To analyze the change of Cdc42 activity after latrunculin A treatment (Figure 2, E and F), image sequences were obtained that included time ranges before and after drug addition. These image sequences were used to generate kymographs along lines that correspond to the direction of migration before latrunculin A addition. An automated ImageJ analysis script was developed to analyze these kymographs. Via this script, the average signal intensity in a range that corresponds to a distance of 0–13 μm from the retracting cell edge was measured for each time point. Signal intensities were normalized to the time point before drug addiction (t = 0 min).

Spatio-temporal image correlation spectroscopy (STICS) flow analysis (Figure 3) was performed using an ImageJ plugin (STICS map jru v2; http://research.stowers.org/imagejplugins/ics_plugins.html). Images of EGFP-Actin were acquired for 15 min with individual frames every 5s. Individual flow vector fields were extracted using 20 frame (i.e. 100s) time intervals. The average flow velocity vector (i.e. “net flow vector” shown in cyan in Figure 3) was calculated by averaging the x and y components of all vectors per cell and then averaging the resulting vectors over time. For each movie, 18 time points were used which were separated by 50s.

For quantification of migration parameters (Figure 4A–E), cells were manually tracked via the ImageJ plugin *Manual Tracking* (Fabrice Cordelires, Institut Curie, Orsay [France]). Migration plots, velocity and distance measurements were computed from these tracks using the chemotaxis tool (Ibidi GmbH, Martinsried, Germany). Directionality was defined as the cell displacement between start and end positions, divided by the travelled migration distance. The number of ectopic back-protrusions was quantified via manual analysis of phase contrast image sequences. Each occurrence of a lamellum or protrusion at a site, at which the cell edge retracted in the previous frame was counted as an ectopic back-protrusion (Figure 4E).

All plotting and statistical analysis were performed using GraphPad Prism 5 software. Figures were assembled using Photoshop CS2014 (Adobe). Stars in the figures denote *p*-values for indicated statistical tests (*, *p* < 0.05; **, *p* < 0.01; ***, *p* < 0.001).

## Abbreviations used:

GBD: GTPase binding domain
GEF: guanine nucleotide exchange factor
MRCKbeta: myotonic dystrophy-related Cdc42-binding kinase beta
NHEKs: normal human epidermal keratinocytes
NMHCIIa: nonmuscle myosin II-A heavy chain
N-WASP: Neural Wiskott-Aldrich syndrome protein
STICS: spatiotemporal image correlation spectroscopy
TIRF-M: total internal reflection fluorescence microscopy
